# Supplementary figures and images for: A map of gene expression in neutrophil-like cell lines
Source: BMC Genomics. 2018 Aug 1;19:573. doi: 10.1186/s12864-018-4957-6 (PMC6090850; doi:10.1186/s12864-018-4957-6)

## Additional File 1

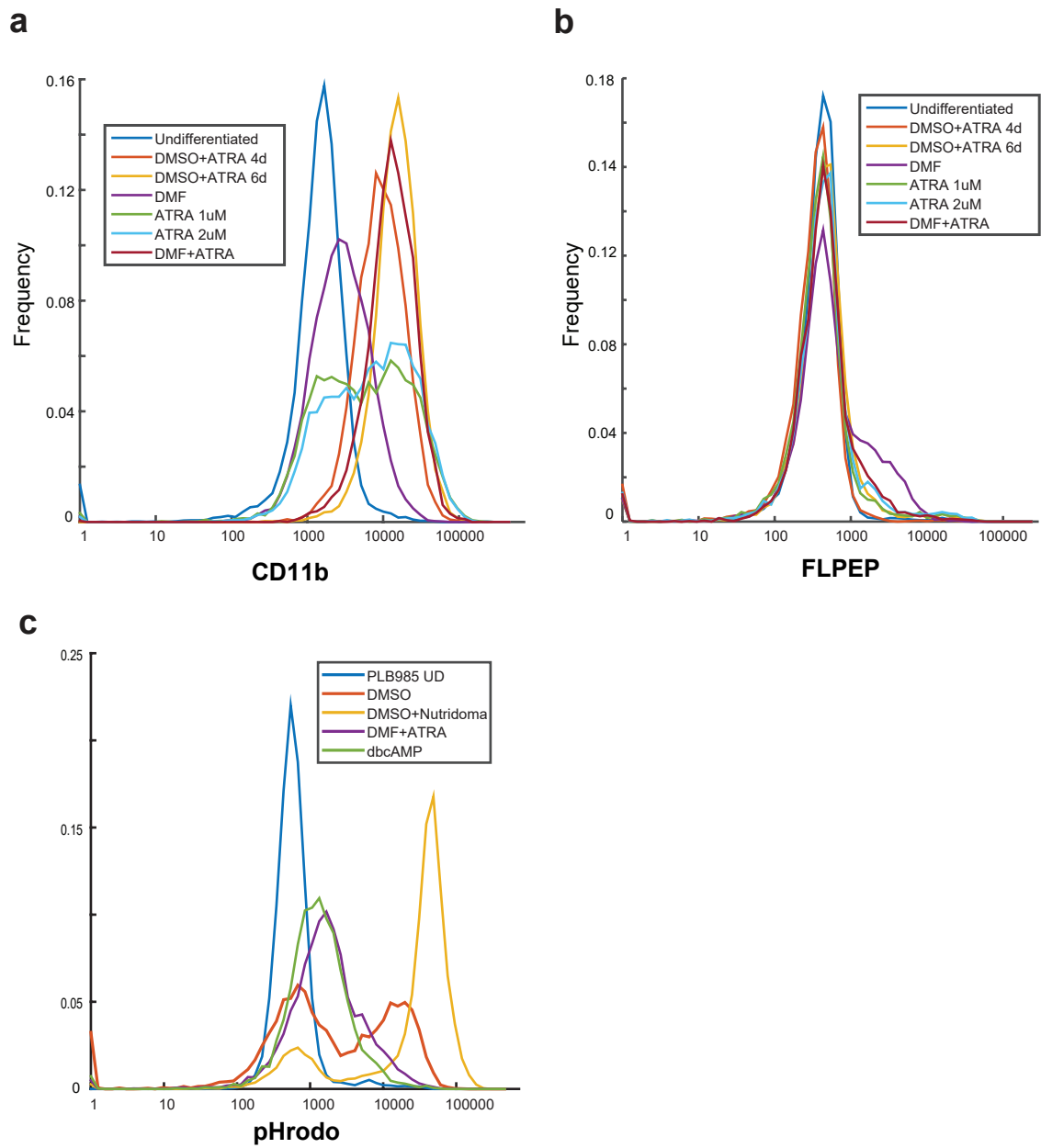

Supplement: Supplementary file 1 — Comparison of cell surface marker expression with different differentiation protocols. PLB-985 cells were differentiated into a neutrophil-like state by culturing in media supplemented with 1.3% DMSO and 2 μM ATRA for 4 days, 1.3% DMSO and 2 μM ATRA for 6 days, 0.5% DMF for 6 days, 1 μM ATRA for 6 days, 2 μM ATRA for 6 days, or 0.5% DMF and 2 μM ATRA for 6 days. Undifferentiated cells were also analyzed. Cells were stained with an antibody against CD11b, chosen as an early differentiation marker (a), or with FLPEP (a fluorescent ligand of FPR1), as a late differentiation marker (b). (c) PLB-985 cells differentiated for 6 days with either 1.3% DMSO, 1.3% DMSO and 2% Nutridoma-CS, 0.5% DMF and 2 μM ATRA, or dbcAMP were mixed in suspension with pHrodo Green-labeled dead Staphylococcus aureus bioparticles for 2 hours at 37 degrees. Phagocytosis of the particles was then analyzed by cytometry and data was analyzed using MATLAB. (PDF 162 kb) [file 12864_2018_4957_MOESM1_ESM.pdf]

Additional File 2

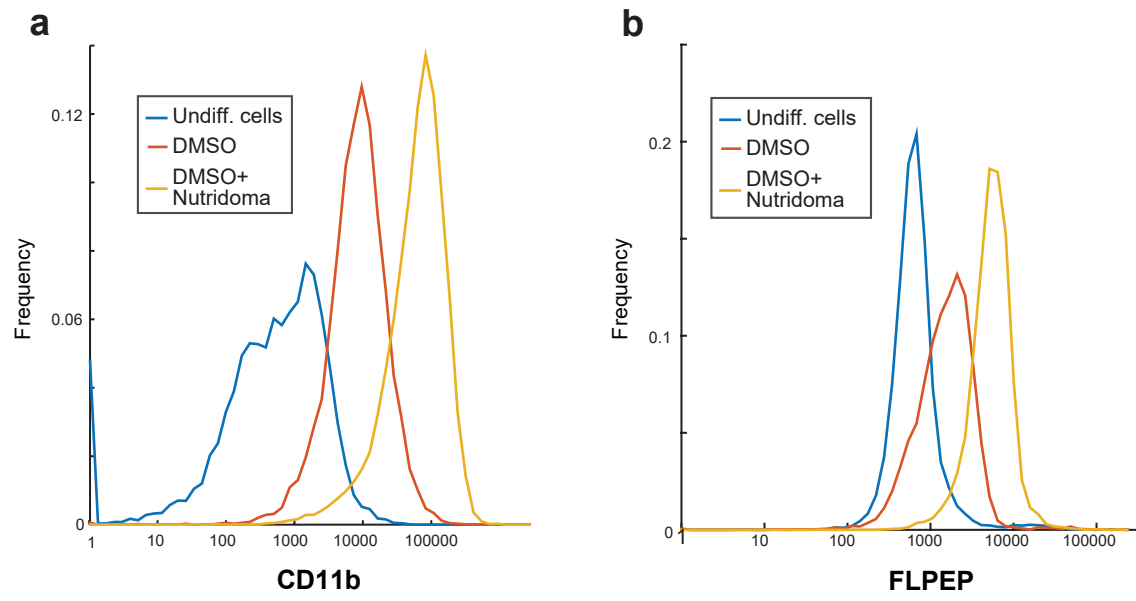

Supplement: Supplementary file 2 — The effect of Nutridoma on surface marker expression is also seen in HL-60 cells. HL-60 cells were differentiated into a neutrophil-like state by culturing in media supplemented with 1.3% DMSO and 9% FBS or supplemented with 1.3% DMSO, 2% Nutridoma and 0.5% FBS, for 6 days. Cells were stained with an antibody against CD11b (a) or the fluorescent FPR1 ligand FLPEP and measured by cytometry. Undifferentiated cells were also analyzed. Data was analyzed using MATLAB. (PDF 134 kb) [file 12864_2018_4957_MOESM2_ESM.pdf]

## Additional File 3

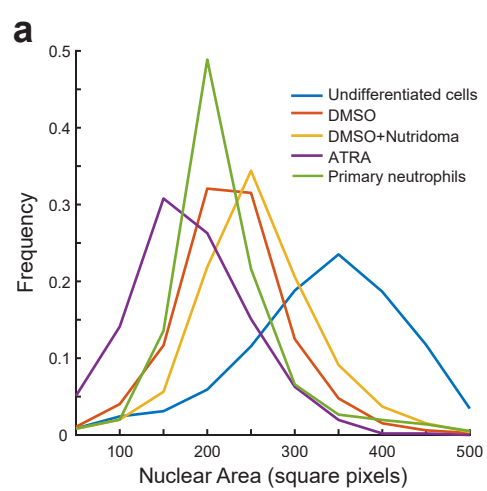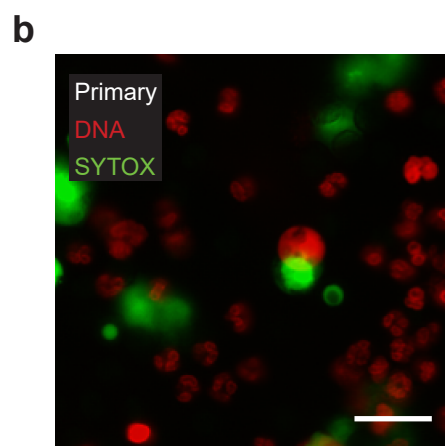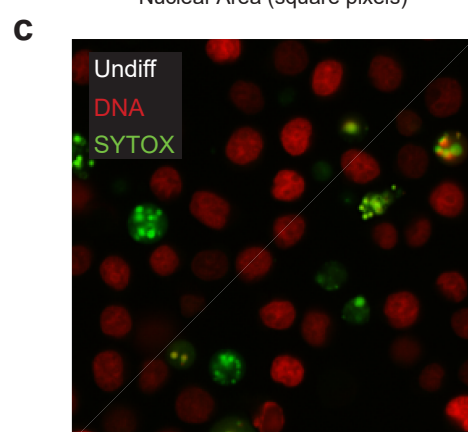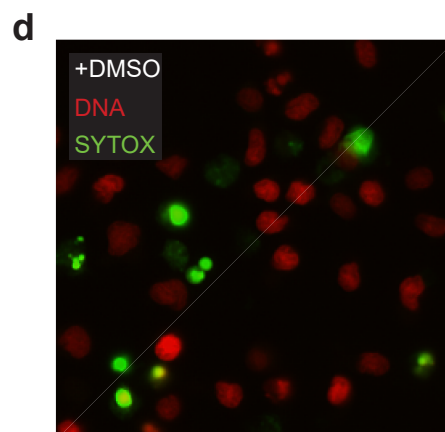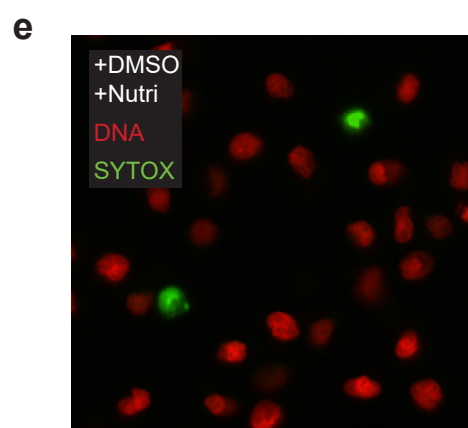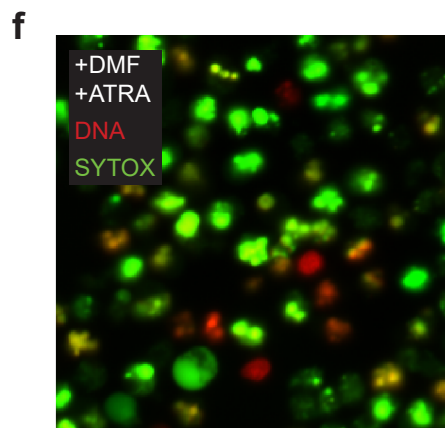

Supplement: Supplementary file 3 — Differentiation of PLB985 cells reduces nuclear area and alters nuclear morphology. (a) Nuclear areas were measured by fluorescence imaging of either histone H2B-mCherry for PLB-985 cells or Hoechst-labeled DNA for primary neutrophils. A histogram of nuclear areas for undifferentiated PLB-985 cells (blue; n = 6203), PLB-985 cells differentiated with DMSO alone (red; n = 4338), PLB-985 cells differentiated with DMSO + Nutridoma CS (yellow; n = 4393), PLB-985 cells differentiated with DMF + ATRA (purple; n = 2386) and human primary neutrophils (green; n = 2264). The X axis represents nuclear area in square pixels, where 1 pixel = 0.4389 μm. Staining with Sytox Blue was used to exclude dead cells from the analysis. (b-f) Representative images of nuclei where scale bar equals 20 μm. DNA is shown in red and Sytox Blue dye (a membrane impermeable DNA-binding dye) is shown in green. (PDF 1850 kb) [file 12864_2018_4957_MOESM3_ESM.pdf]

Additional File 4

**a**

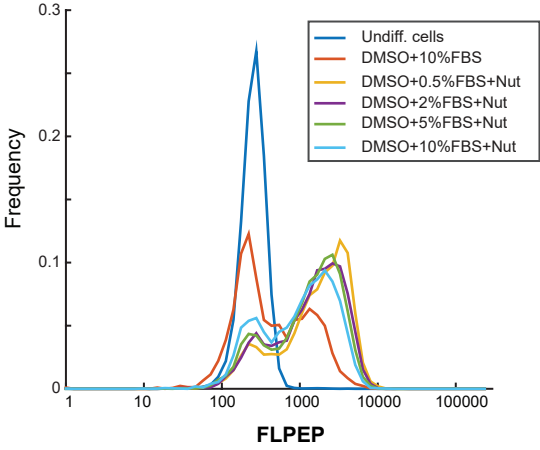

**b**

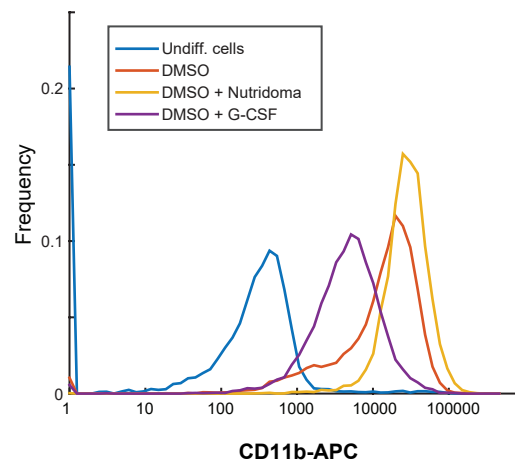

**c**

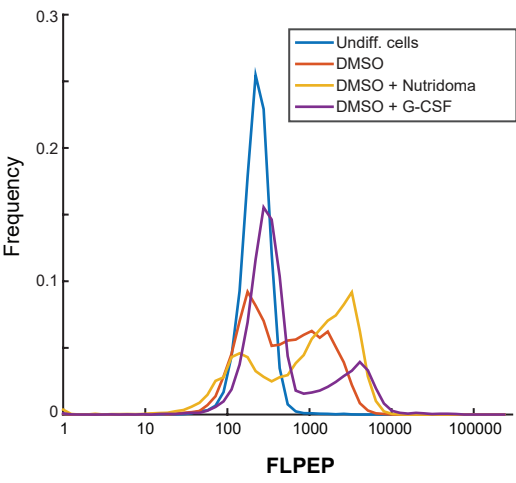

Supplement: Supplementary file 4 — (a) PLB-985 cells were differentiated into a neutrophil-like state by culturing in media supplemented with 1.3% DMSO, 2% Nutridoma and the indicated percentage of FBS. Cells were stained with FLPEP and measured by cytometry. (b-c) PLB-985 cells were differentiated into a neutrophil-like state by culturing in media supplemented with 1.3% DMSO 0.5% FBS and either 2% Nutridoma or 30 ng/mL G-CSF for 6 days. Cells were stained with anti-CD11b (b) or with FLPEP (c) and measured by cytometry. Cytometry data was analyzed using MATLAB in all the experiments. Data for the undifferentiated cells and those differentiated with DMSO or DMSO + Nutridoma is the same as that shown in Fig. 2. (PDF 158 kb) [file 12864_2018_4957_MOESM4_ESM.pdf]

## Additional File 7

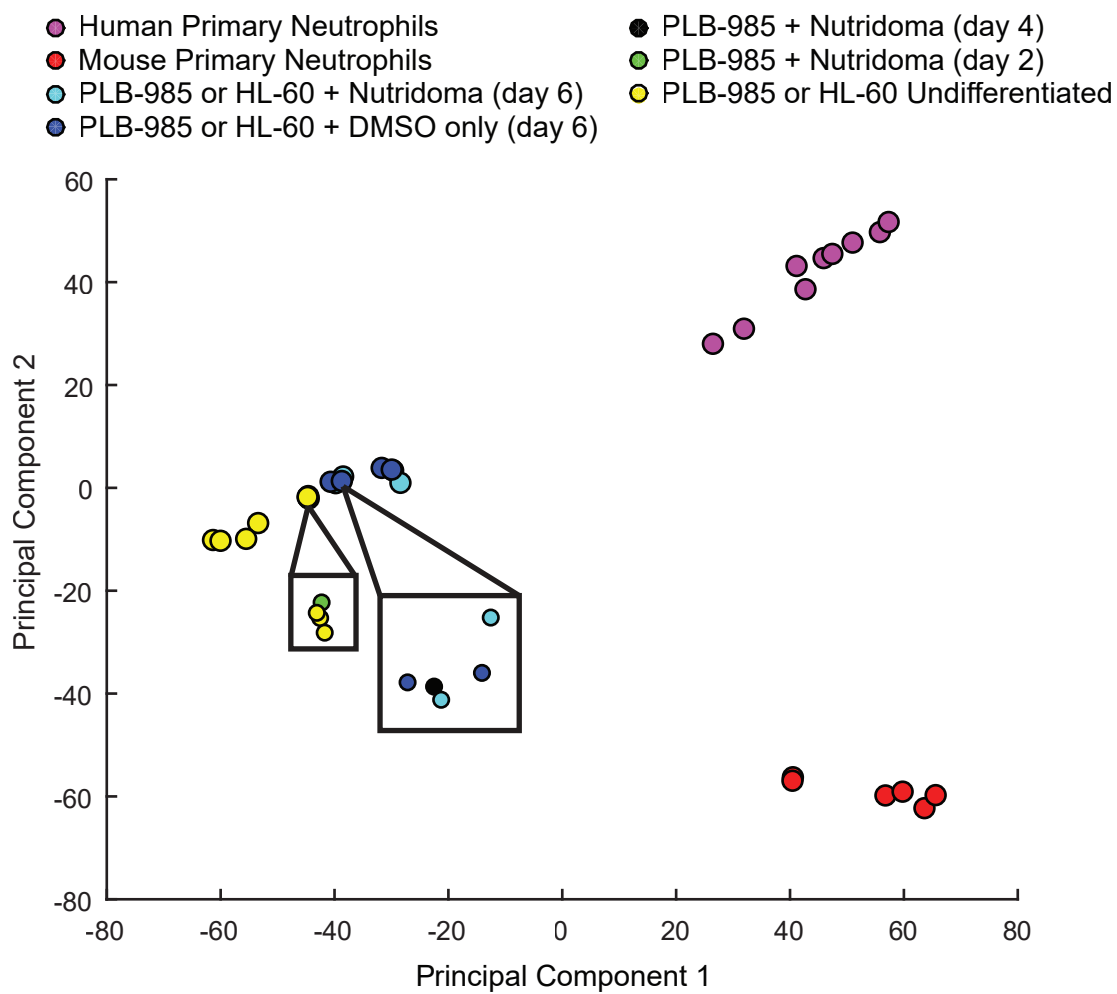

Supplement: Supplementary file 7 — Principal component analysis of the gene expression data analyzed in this study. Principal component analysis was carried out using MATLAB’s “pca” function with the default options for all data sets included in this study (the same data sets that are included in Fig. 4b). Only log10 expression values for genes with both human and mouse homologs were included for the analysis. To avoid a large influence from noise in lowly expressed genes, all log10 expression below − 2 were set to − 2 for this analysis. Shown is a scatter plot of the first two principal components. Together, these two components explained 66% of the variance in the data set. (PDF 357 kb) [file 12864_2018_4957_MOESM7_ESM.pdf]

## Additional File 8

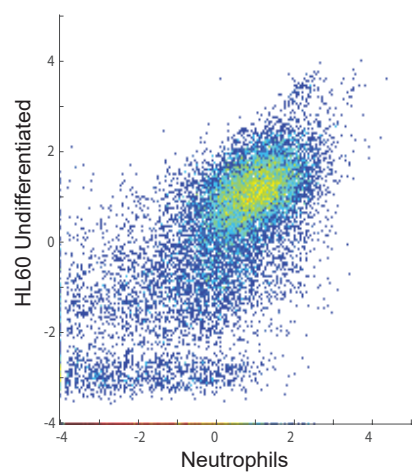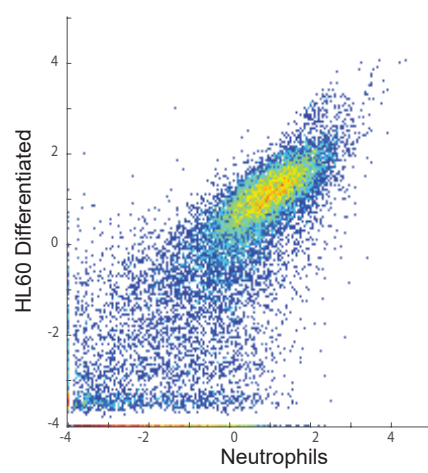

Supplement: Supplementary file 8 — Similarity of gene expression between HL-60 cells and primary human neutrophils. Density-colored scatter plots of expression values (normalized FPKM on a log10 scale) for protein-coding genes for primary human neutrophils versus undifferentiated HL-60 cells (left) or those differentiated with DMSO + Nutridoma (right). (PDF 364 kb) [file 12864_2018_4957_MOESM8_ESM.pdf]

Additional File 9

**a**

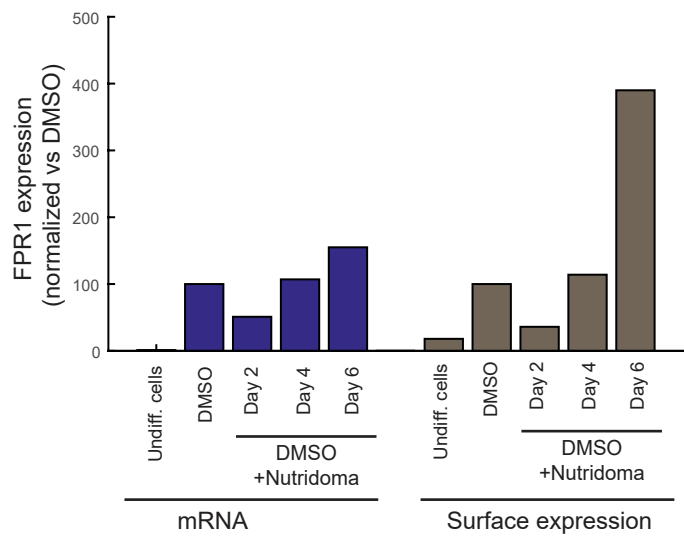

**b**

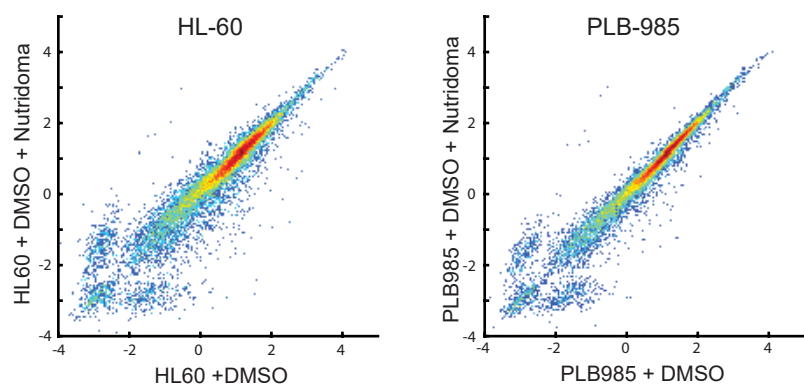

**c**

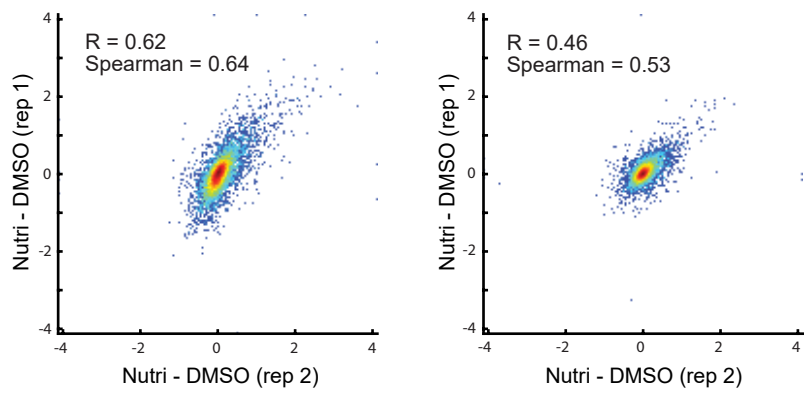

Supplement: Supplementary file 9 — Nutridoma affects gene expression during differentiation subtly, but consistently in HL-60 and PLB-985 cells. (a) PLB-985 cells were differentiated into a neutrophil-like state by culturing in media supplemented with 1.3% DMSO, 2% Nutridoma and 0.5% FBS. At the indicated days post-differentiation, cells were harvested and processed for RNA-seq analysis or stained with FLPEP. Shown are the expression level of FPR1 at the mRNA level (from our RNA-seq data) and at the surface of the cells (FLPEP staining). Values for undifferentiated cells and for cell differentiated with 1.3% DMSO and 9% FBS are shown for comparison. Values were normalized by the values for the sample differentiated with only DMSO. (b) Density-colored scatter plots of expression values (normalized FPKM on a log10 scale) for protein-coding genes for HL-60 (left) or PLB-985 cells (right) cells differentiated with DMSO + Nutridoma versus those differentiated with DMSO. These data represent the averages of two replicate experiments. (c) Density-colored scatter plots of log10 fold-changes between the two protocols (subtracting DMSO values from DMSO + Nutridoma) comparing each of our two replicates of the RNA-Seq experiments for HL-60 (left) or PLB-985 (right) cells. To avoid noise from lowly expressed genes, only protein-coding genes with a mean log10 normalized expression value of − 1 or higher were included. The Pearson’s (R) and Spearman’s correlation coefficients are shown. (PDF 417 kb) [file 12864_2018_4957_MOESM9_ESM.pdf]

Additional File 11

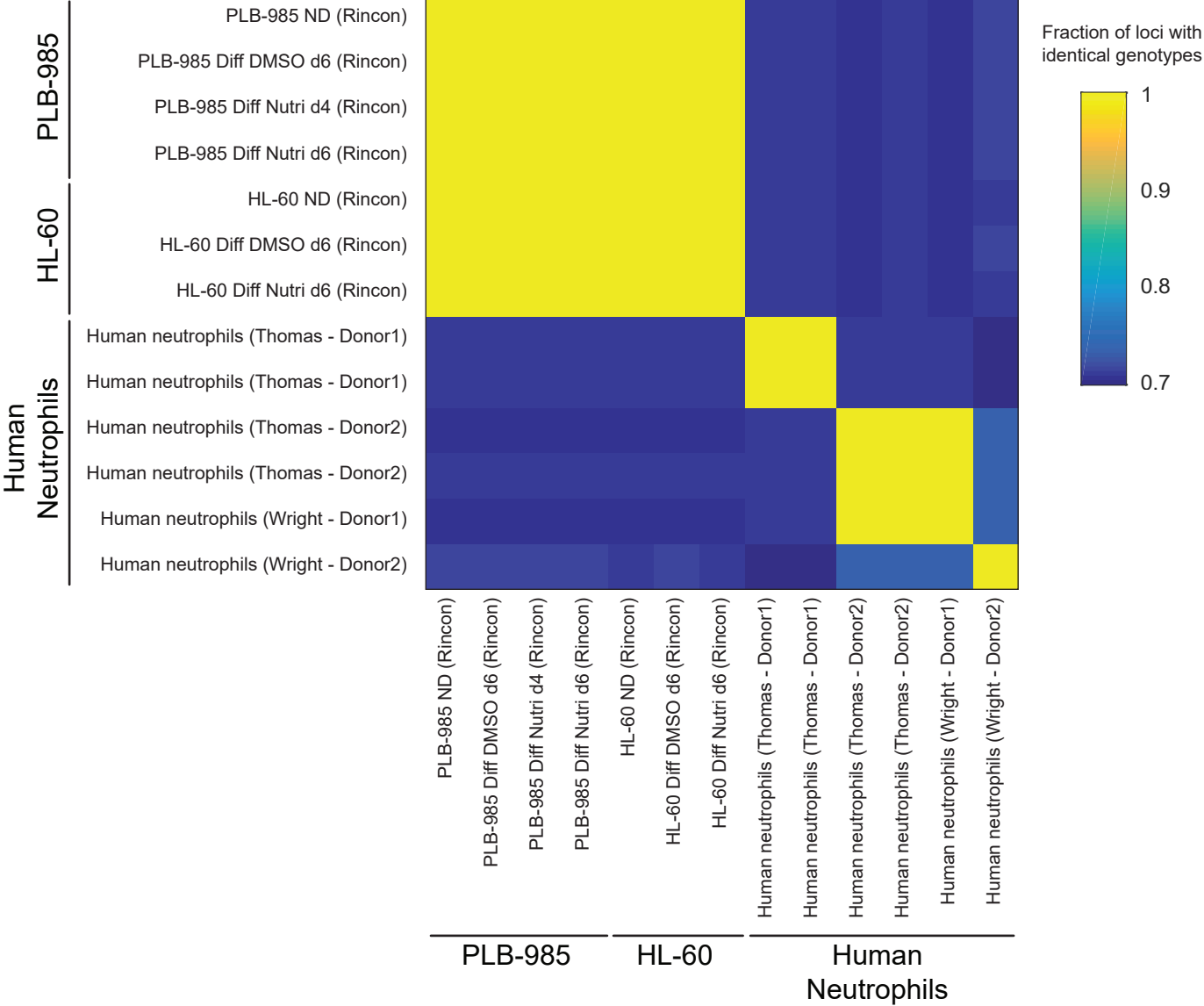

Supplement: Supplementary file 11 — SNP analysis confirms that PLB-985 is genetically identical to HL-60. Variant calling for genotyping was performed using the GATK pipeline and the GATK best practices guidelines for each sample from our RNA-Seq raw data and from the primary human neutrophil RNA-Seq data. The heatmap indicates the fraction of loci with identical genotypes for each pair of samples (see Methods for more details). (PDF 377 kb) [file 12864_2018_4957_MOESM11_ESM.pdf]
